# 3D-assisted arthroscopic management of subacromial impingement: A clinical outcome study

**DOI:** 10.1051/sicotj/2026065

**Published:** 2026-10-21

**Authors:** Quyet Tran, Dung Trung Tran, Thuong Van Pham, Hiep Dai Tran, Trung Quoc Nguyen

**Affiliations:** 1 Center of Orthopaedic and Sport Medicine, Vinmec Healthcare System Hanoi Vietnam; 2 College of Health Sciences, VinUniversity Hanoi Vietnam; 3 Vinmec – VinUni Institute of 3D Technology in Medicine Hanoi Vietnam; 4 Hai Phong University of Medicine and Pharmacy Haiphong Vietnam

**Keywords:** Acromioplasty, Subacromial impingement, 3D technology, Arthroscopic treatment

## Abstract

*Introduction*: The clinical value of three-dimensional (3D)–assisted planning in arthroscopic acromioplasty remains unclear, particularly regarding its ability to optimize subacromial decompression and improve clinically meaningful outcomes. *Purpose*: To evaluate whether 3D-assisted preoperative planning can improve radiologic parameters and functional outcomes in patients undergoing arthroscopic treatment for subacromial impingement syndrome. *Study design*: Descriptive observational study (combined retrospective and prospective). *Methods*: Fifty patients with subacromial impingement syndrome underwent arthroscopic acromioplasty with 3D-assisted preoperative planning. Clinical outcomes were assessed using the UCLA score. Radiologic parameters, including acromial index (AI), critical shoulder angle (CSA), lateral acromial angle (LAA), and acromiohumeral distance (AHD), were evaluated using static and dynamic 3D computed tomography preoperatively and postoperatively. *Results*: Postoperatively, AI and CSA significantly decreased toward normal ranges, while LAA improved. Both static and dynamic AHD increased significantly, indicating enhanced subacromial space. The mean UCLA score improved from 21.54 ± 2.66 to 33.22 ± 1.75 (*p* < 0.01). *Conclusion*: 3D-assisted arthroscopic acromioplasty was associated with significant improvements in both radiologic parameters and functional outcomes. The ability to evaluate subacromial space dynamically and localize impingement sites may facilitate more precise and individualized decompression, potentially enhancing surgical effectiveness.

## Introduction

With the growing trend toward minimally invasive and personalized surgical approaches, there is still a lack of studies evaluating the outcomes of applying 3D technology in surgery. Notably, Yoshida et al. [1] and Werner et al. [2] reported that three-dimensional measurements of the acromiohumeral distance were smaller than those obtained using two-dimensional methods. Subsequent studies comparing the reliability of shoulder anatomical measurements suggested that 3D reconstruction techniques minimize measurement errors and yield greater accuracy than 2D assessments in evaluating the scapulohumeral head distance [3–5]. Therefore, we conducted the study entitled “3D-Assisted Arthroscopic Management of Subacromial Impingement: A Clinical Outcome Study” with the primary objective of evaluating the clinical outcomes of arthroscopic management of subacromial impingement using 3D technology in patients with subacromial impingement. With the application of CT and three-dimensional (3D) reconstruction, the location of subacromial space narrowing can be quantitatively assessed under both static and dynamic conditions. Dynamic simulation of glenohumeral motion, including forward elevation and shoulder abduction, enables identification of the acromial region responsible for the greatest reduction in the subacromial space during functional movement. In addition, 3D reconstruction provides intuitive visualization of acromial morphology and facilitates quantitative localization of the region of maximal subacromial narrowing. These findings can be incorporated into preoperative planning to determine the target site and estimated extent of acromial bone resection, thereby enabling a more individualized arthroscopic acromioplasty than conventional imaging alone (Figure 1).

**Figure 1:**
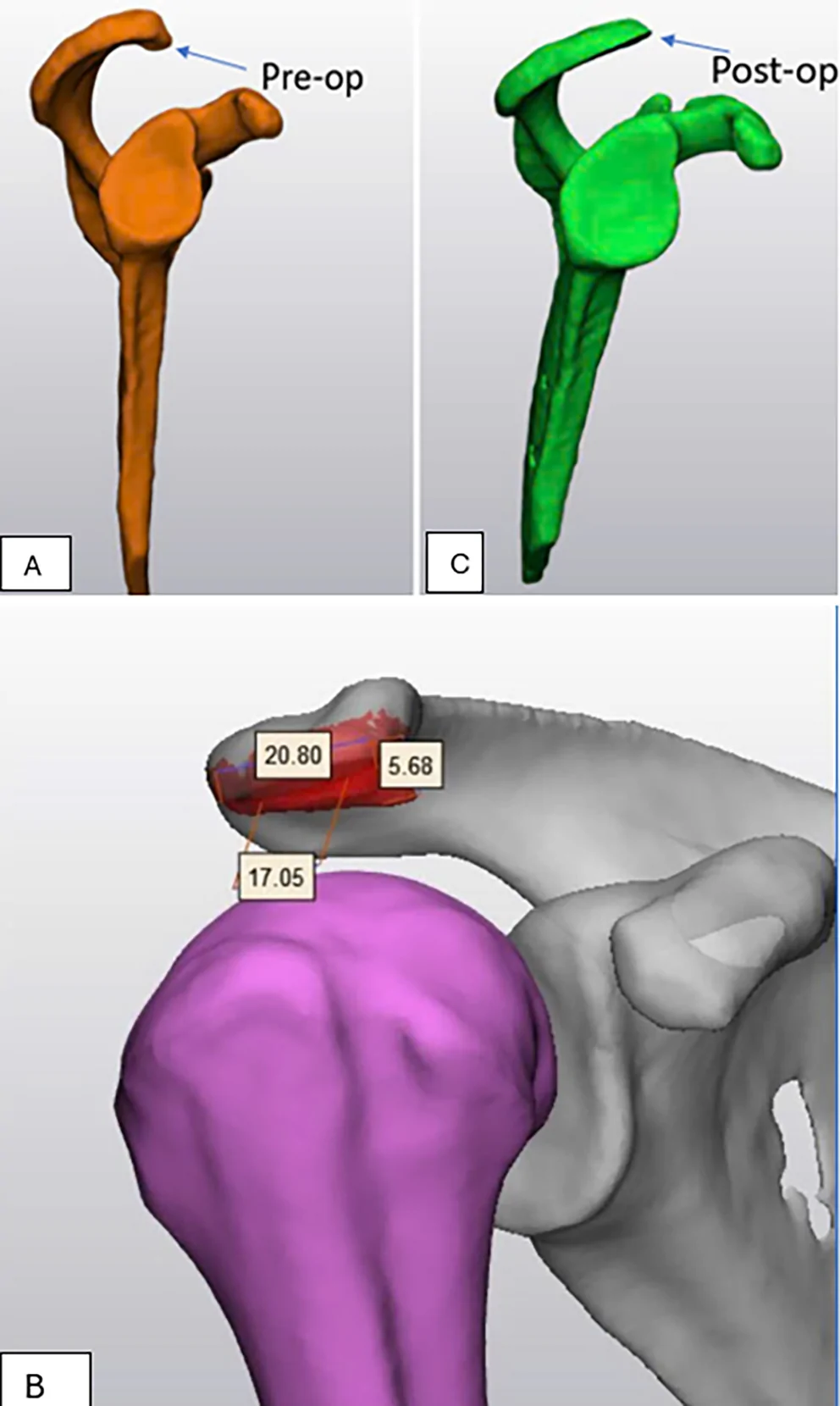
Preoperative and postoperative acromial morphology (A, C); The red-colored region represents the area responsible for subacromial impingement, together with the dimensional parameters of the planned acromioplasty (B).

## Materials and methods

### Study population

A total of 50 patients with subacromial impingement, with or without associated rotator cuff pathology, who were diagnosed and surgically treated at our hospital from January 2023 to June 2025.

### Study design

A descriptive study combining retrospective and prospective components. Patients were clinically evaluated preoperatively and at 1 month, 3 months, 6 months, and 1 year postoperatively using the UCLA shoulder score. In terms of imaging, all patients underwent computed tomography (CT) in both static and dynamic positions. The assessed parameters included the Acromial Index (AI), Critical Shoulder Angle (CSA), Acromiohumeral Distance (AHD), and acromial morphology. The indication for arthroscopic acromioplasty was established based on the patient's clinical presentation, physical examination, MRI findings, and conventional radiographs. Preoperative 3D CT reconstruction was subsequently performed for surgical planning rather than for determining surgical indication. Dynamic simulation of shoulder motion was used to identify the region of maximal subacromial narrowing and to quantify the minimum acromiohumeral distance. These findings were used to preoperatively determine the location and estimated thickness of acromial bone resection. Standard arthroscopic portal placement was used in all cases, while intraoperative bone removal was guided by preoperative 3D planning.

### Inclusion criteria

- Adult patients (≥18 years), regardless of sex.
- Patients with a confirmed diagnosis of subacromial impingement syndrome based on clinical evaluation (positive Neer test, Hawkin–Kennedy test, Impingement test) and imaging, including 3D computed tomography (CT), magnetic resonance imaging (MRI), and plain radiography (X-ray), including those with or without rotator cuff tears.
- Patients undergoing arthroscopic acromioplasty with preoperative 3D CT–based planning to determine the site of maximal subacromial narrowing, including both isolated acromioplasty and cases combined with rotator cuff repair.
- Patients who agreed to participate in the study.

### Exclusion criteria

- Patients with contraindications to surgery due to medical comorbidities or inability to undergo general anesthesia with endotracheal intubation.
- Patients with a postoperative follow-up period of less than 3 months.
- Patients with incomplete medical records.
  - Sample size: A total sampling approach was applied.
  - Statistical analysis was performed using SPSS 21.
  - Continuous variables were expressed as mean ± standard deviation. Preoperative and postoperative values were compared using paired *t*-tests for normally distributed data. A *p*-value <0.05 was considered statistically significant.
  - Follow-up protocol: Patients were scheduled for follow-up visits at 1, 3, 6, and 12 months postoperatively. At each follow-up, clinical examination, functional assessment, and radiographic evaluation were performed. Functional outcomes were assessed using the UCLA Scale. Standard anteroposterior and outlet-view radiographs were obtained to evaluate the acromial morphology and detect any postoperative complications. Patients who were unable to attend outpatient follow-up were contacted by telephone to complete the functional assessment whenever applicable. All patients completed the minimum 12-month follow-up. No patients were lost to follow-up during the study period, and all patients completed the scheduled postoperative evaluations.
  - All procedures were performed by a single experienced orthopedic surgeon using a standardized arthroscopic acromioplasty technique based on the preoperative 3D CT planning protocol. The same surgical protocol was applied in all patients to minimize inter-procedural variability.
  - Three-dimensional anatomical models of the scapula, humerus, and implant were reconstructed from each patient's CT data. After segmentation and 3D reconstruction, the models were imported into Mimic software for kinematic analysis of the shoulder joint. Anatomical coordinate systems were established for the scapula and humerus based on standardized anatomical landmarks. The humerus was allowed to rotate about the center of the glenohumeral joint to simulate physiological shoulder motions, including forward flexion, extension, abduction, and abduction with external rotation. For each motion, the humerus was sequentially positioned at predefined joint angles. Forward flexion was simulated at 30°, 60°, 90°, 120°, and 150°; extension at 30°, 60°, and 90°; abduction at 30°, 60°, 90°, and 120°; and abduction with external rotation at 30°, 60°, and 90°. At each position, the model was temporarily fixed to allow measurement. For example, in the measurement of the acromiohumeral distance (AHD), the value was automatically calculated using a surface-to-surface minimum distance algorithm within the three-dimensional simulation environment. To ensure measurement consistency, all analyses were performed using the same anatomical coordinate system and the same 3D model dataset.

## Results

### Preoperative acromial morphology on 3D CT

All cases in the study group demonstrated acromial osteophytes on 3D CT reconstruction, accounting for 100%.

### Preoperative acromial morphology and rotator cuff characteristics on MRI

All patients demonstrated acromial osteophytes and subacromial space narrowing on preoperative MRI; 80.0% had rotator cuff tears, while 20% had isolated impingement without rotator cuff tear.

## Discussion

Arthroscopic subacromial decompression and acromioplasty are among the most performed procedures in shoulder arthroscopy. These techniques are indicated for cases of persistent subacromial bursitis and subacromial impingement syndrome. The aim of the procedure is to remove the subacromial bursa and the osteophytes on the undersurface of the acromion.

Theoretically, the acromion and the coracoacromial ligament form an arch that conforms to the curvature of the humeral head. Codman [6] further suggested that anterior osteophytes represent an extension of the attachment of the coracoacromial ligament and are not necessarily pathological. In this study, none of the patients demonstrated abnormal bone tumors of the shoulder joint or acromion, nor were there signs of glenohumeral or acromioclavicular joint degeneration. All patients in the study group exhibited calcification on the undersurface of the anterolateral acromion, which is an indirect sign suggestive of subacromial impingement (Table 1).

The Acromial Index (AI) was first described by Nyffeler et al. [7]. It is measured on a standardized anteroposterior radiograph by calculating the ratio of the distance from the glenoid plane to the lateral edge of the acromion to the distance from the glenoid plane to the lateral edge of the humeral head. A higher AI shifts the deltoid force vector more vertically, requiring the rotator cuff to generate greater horizontal force to stabilize the humeral head during arm abduction. Conversely, a lower AI results in a more compressive force of the deltoid on the glenoid, acting synergistically with the rotator cuff.

Compared with the original study by Nyffeler et al., in which the AI was reported as 0.73 ± 0.06, the values reported by Miyazaki and Fregoneze were 0.7194 [8]. Thus, preoperatively, all cases demonstrated an Acromial Index (AI) higher than the normal range, consistent with the patients’ clinical symptoms. The mean AI decreased from 0.82 ± 0.03 before surgery to 0.58 ± 0.04 after surgery (Figure 2), which is lower than the values reported in the aforementioned studies.

**Figure 2:**
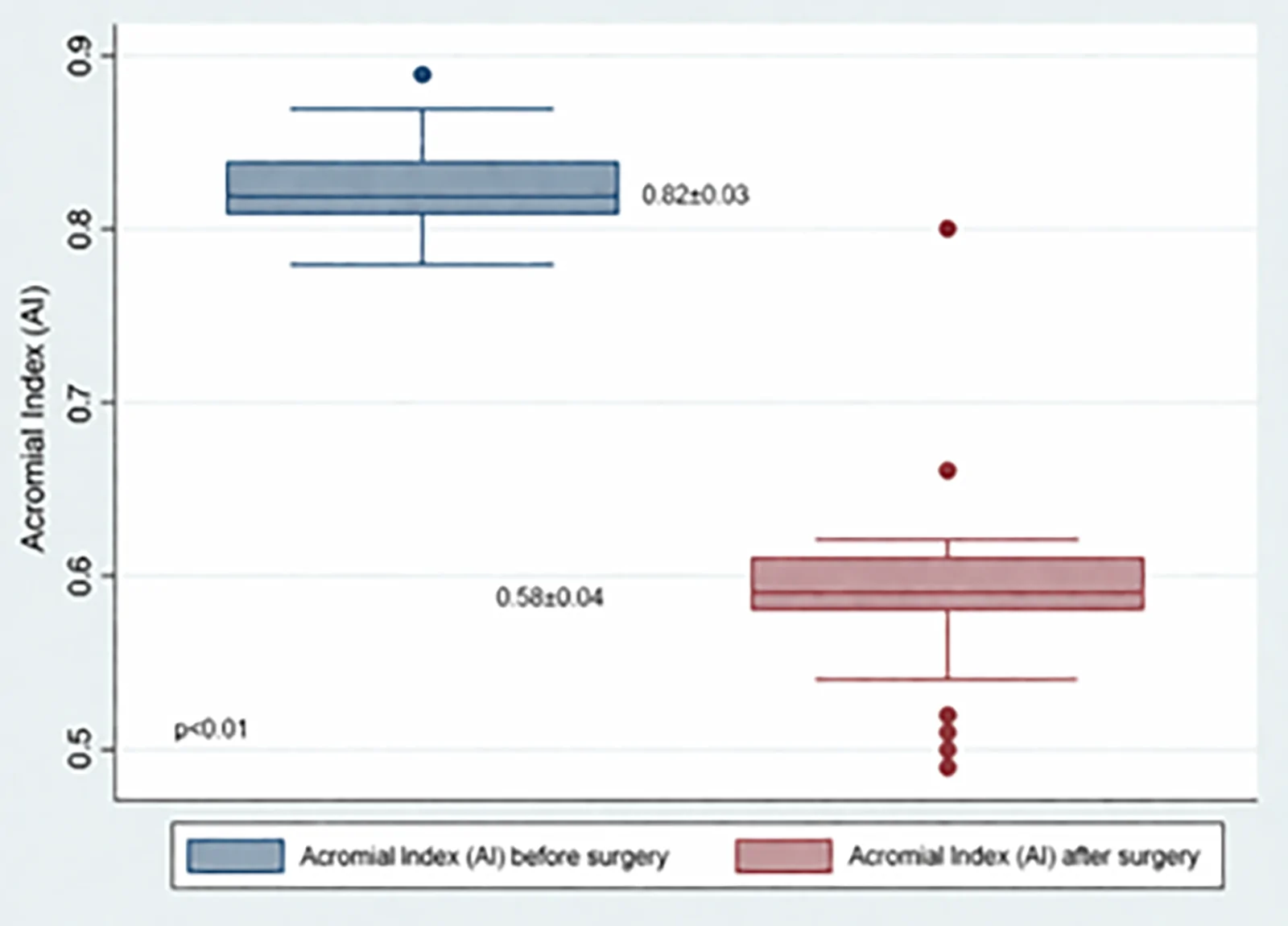
Acromial Index (AI) before and after surgery (blue is before surgery and pink is after surgery). The preoperative Acromial Index (AI) was 0.82 ± 0.03, which decreased to 0.58 ± 0.04 postoperatively; the difference was statistically significant (*p* < 0.01).

Moor et al [9] proposed to account for two aspects: the lateral extension of the acromion and the inclination of the glenoid. A more superiorly inclined glenoid facilitates upward translation of the humeral head by the deltoid, thereby requiring the rotator cuff to generate greater force to stabilize the humeral head. Biomechanical studies have shown that a higher CSA increases the load on the supraspinatus tendon in maintaining humeral head stability during active abduction and is associated with a higher risk of primary rotator cuff tears and postoperative re-tears. This mechanism is thought to contribute more significantly to rotator cuff injury than the abrasion mechanism [8, 10, 11]. Optimal CSA ranges 30–35° [12]. A CSA >35° increases shear forces on the rotator cuff, whereas a CSA <30° may lead to glenohumeral osteoarthritis due to increased compressive forces across the joint. [13]. In our study, the preoperative CSA was 38.29 ± 0.63, which decreased to 33.33 ± 1.17 postoperatively (Figure 3), falling within the normal range and comparable to the results reported in previous studies. Thus, with the support of 3D technology in preoperative planning, we were able to reduce the CSA by approximately 5.5–6.7°, representing a greater improvement compared with unplanned anterior or lateral acromioplasty, which typically achieves a reduction of only about 3° to 4° [14].

Acromial morphology on the sagittal plane, based on the classification by Bigliani et al., is divided into three types: type I (flat), type II (curved), and type III (hooked). In the study by Azalia [15] regarding the relationship between acromial morphology and shoulder pathology, Azalia reported that type II acromion was the most common and found no significant correlation between acromial shape and shoulder disorders. However, in our study, preoperative results showed that no patients had type I acromion. Most subjects had a type II acromion (77.6%), while 22.4% had a type III acromion (Table 2). Postoperative results showed that 100% of patients had a type I acromion, thereby minimizing the risk of future subacromial impingement.

**Table 1:** Preoperative MRI findings (*n* = 50).

Characteristics		Number	Percentage %
Acromial osteophyte	Yes	50	100.0
	No	0	0.0
Subacromial impingement	Yes	50	100.0
	No	0	0.0
Rotator cuff tear	Yes	40	80.0
	No	10	20.0
Total		**50**	**100.0**

**Table 2:** Acromial morphology on 3D CT.

Type	Numbers	Percentage %
Type 1	0	0.0
Type 2	39	78.0
Type 3	11	22.0
Total	50	100

No patients had type I acromion. The majority of subjects had a type II acromion (77.6%), while 22.4% had a type III acromion.

**Table 3:** Preoperative and postoperative UCLA (*n* = 50).

UCLA	Mean ± SD	Min–max	*p*
Preoperation	21.54 ± **2.66**	18–27	<0.01*
Postoperation	33.22 ± **1.75**	28–35	

The mean preoperative UCLA score was 21.54 ± 2.66, which increased to 33.22 ± 1.75 postoperatively, indicating marked improvement in clinical and functional outcomes. The difference was statistically significant (*p* < 0.01).

The mean acromiohumeral distance (AHD) in the absence of rotator cuff pathology is approximately 10.5 mm [16], from 6 to 14 mm, as reported by Cotton and Rideout [17] or from 7 to 13 mm, as reported by Weiner and Macnab [18]. An acromiohumeral distance (AHD) ≤7 mm may indicate a rotator cuff tear, with a specificity of approximately 75% [19] and is also a predictive factor for re-tear after rotator cuff repair [20]. Following surgery, the mean acromiohumeral distance (AHD) in the static position on 3D CT increased from 5.26 ± 0.65 mm preoperatively to 8.77 ± 1.03 mm postoperatively (Figure 3), which falls within the normal range compared with previous studies. Additionally, the mean AHD in the dynamic position on 3D CT increased from 0.96 ± 0.56 mm preoperatively to 4.16 ± 0.49 mm postoperatively, also within the normal range.

### LAA

The Lateral Acromial Angle (LAA) is defined as the angle formed between a line along the undersurface of the acromion and the plane of the glenoid. Previous studies have shown that a lower LAA is associated with subacromial impingement, particularly during arm elevation.

In our study, the mean LAA in patients with subacromial impingement (including both those with and without rotator cuff tears) was 61.47° ± 2.26°, which is markedly lower than the normal range reported in prior studies. Banas et al. (1995) reported a mean LAA of approximately 78° (range 64–99°), and notably, all cases with an LAA ≤70° were associated with full-thickness rotator cuff tears [21]. Several authors have suggested that a lower threshold of LAA in the range of 70–75° may help reduce impingement on the rotator cuff tendons. In addition, Wu et al. found an association between LAA and the onset of postoperative symptoms in patients undergoing arthroscopic rotator cuff repair [22]. Several studies have confirmed that a low LAA is strongly associated with subacromial impingement syndrome and rotator cuff tears and is considered an independent anatomical risk factor.

Compared with the literature, the LAA in our study (approximately 61°) (Figure 4) is not only lower than the previously reported average values but also falls well within the high-risk range (<70°) [23]. These findings suggest that the acromial morphology in Vietnamese patients in our cohort may play an important role in the pathogenesis, contributing to a higher incidence of rotator cuff tears. This result also indicates that the LAA may serve as a useful parameter for screening and predicting the risk of injury in patients presenting with clinical symptoms of impingement syndrome.

**Figure 4:**
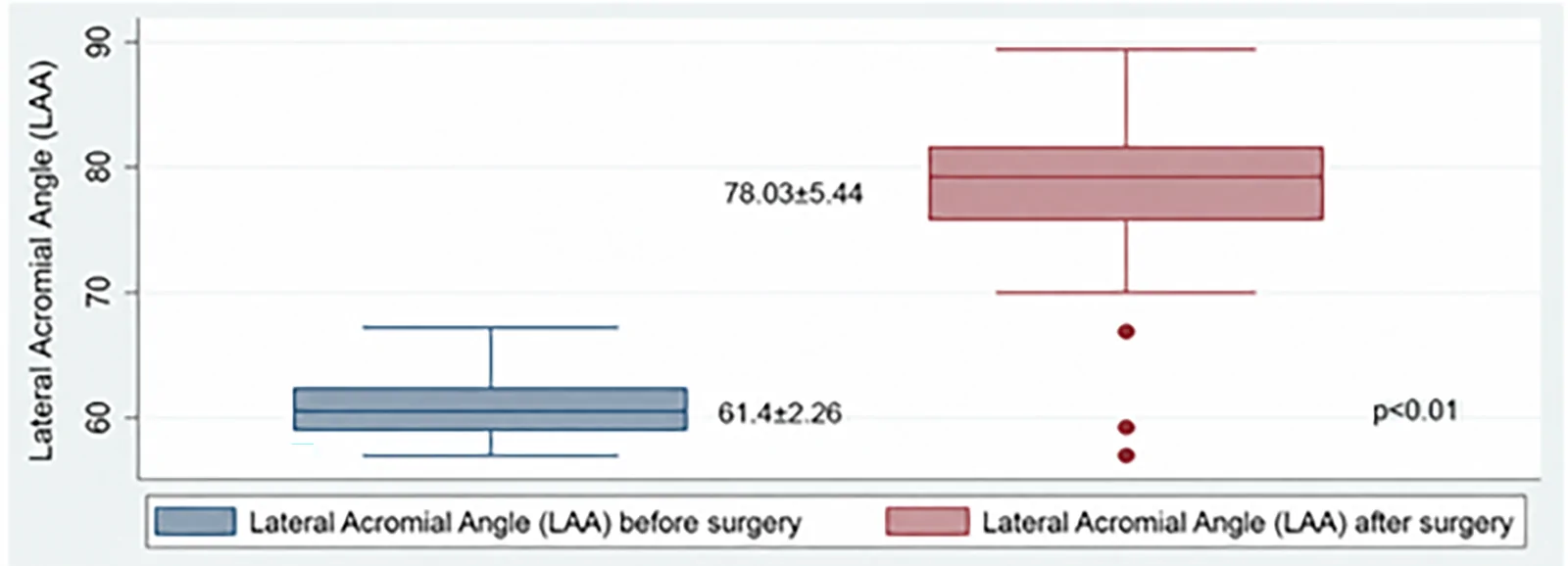
LAA before and after surgery (blue is before surgery and pink is after surgery). The preoperative Lateral Acromial Angle (LAA) was 61.4 ± 2.26, which increased to 78.03 ± 5.44 postoperatively; the difference was statistically significant (*p* < 0.01).

**Figure 3:**
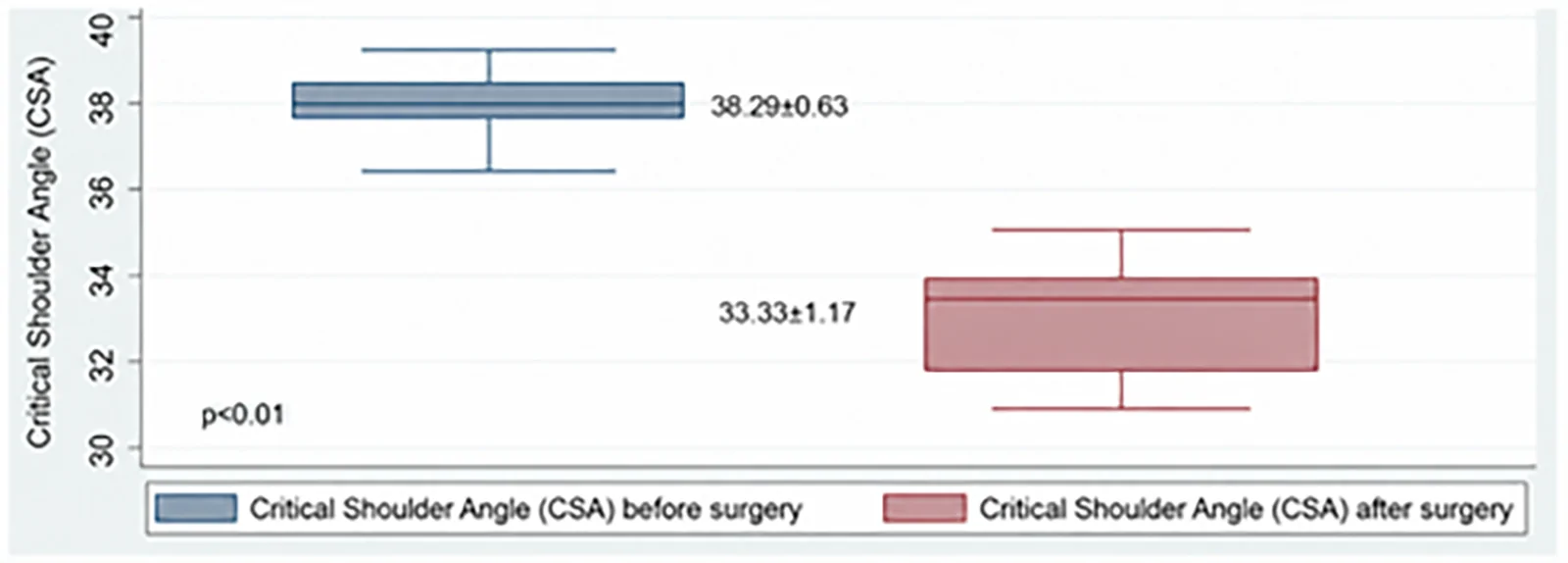
CSA before and after surgery (blue is before surgery and pink is after surgery). The preoperative Critical Shoulder Angle (CSA) was 38.29 ± 0.63, which decreased to 33.33 ± 1.17 postoperatively; the difference was statistically significant (*p* < 0.01).

**Figure 5:**
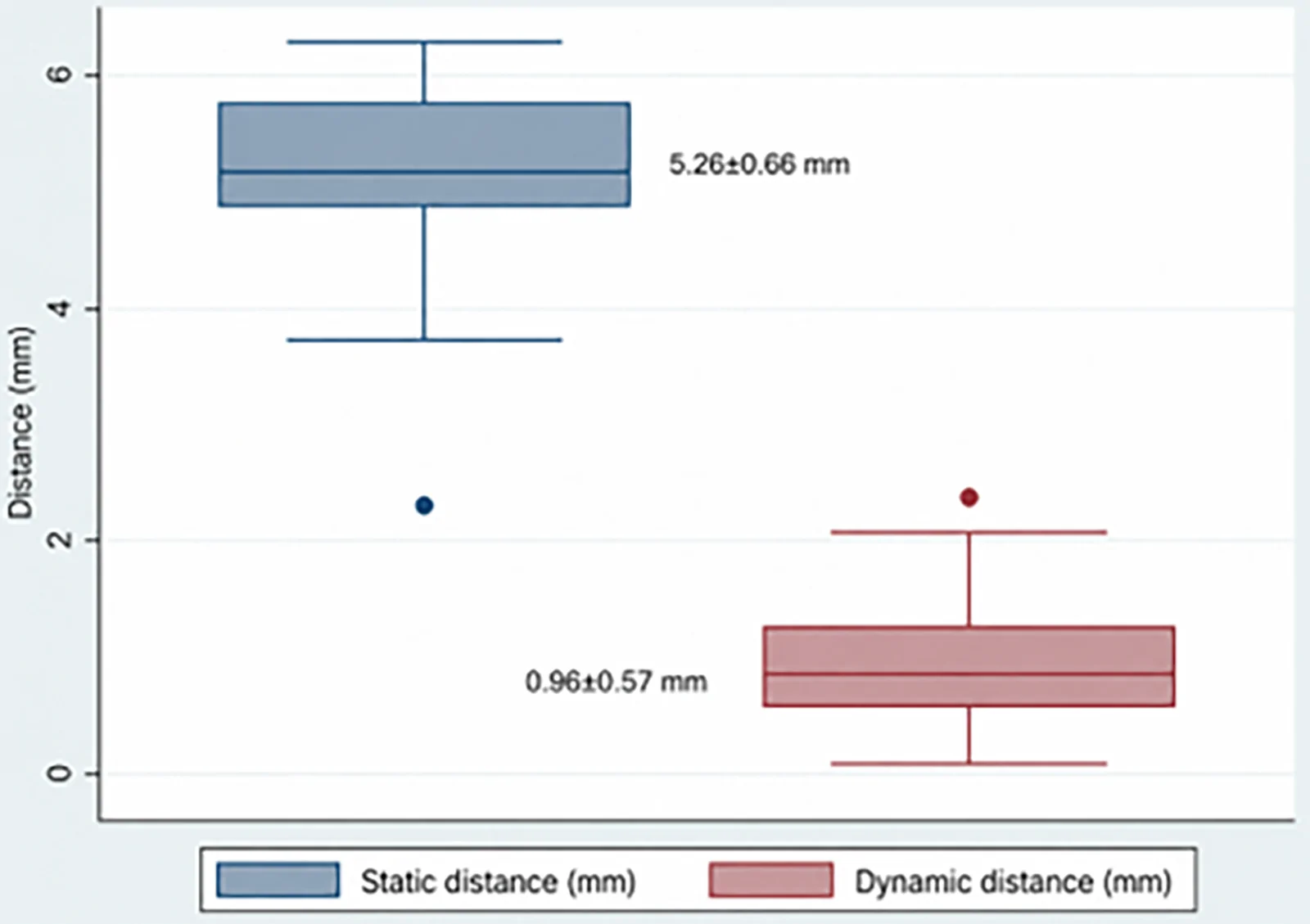
Mean acromiohumeral distance (AHD) in the static position (blue) and in the dynamic position measured through simulated motion (pink) on preoperative 3D CT (*n* = 50). The mean acromiohumeral distance (AHD) in the static position on preoperative 3D CT was 5.26 ± 0.66 mm, whereas the mean AHD in the dynamic position, measured through simulated motion on 3D CT, was 0.96 ± 0.57 mm.

Therefore, our study adds further evidence supporting the important role of the LAA in the evaluation and management of shoulder disorders.

### Mean acromiohumeral distance (AHD) in static and dynamic positions on 3D CT

In our study, the mean acromiohumeral distance (AHD) in the static position (3D CT) was 5.26 ± 0.66 mm, which decreased markedly in the dynamic position (simulated motion on 3D CT) to only 0.96 ± 0.57 mm. This extremely small distance clearly reflects the reduction of the subacromial space during shoulder motion, particularly during arm elevation.

Previous studies have also demonstrated a progressive narrowing of the subacromial space (AHD) with arm elevation, with greater reductions observed at higher degrees of motion. For example, dynamic simulations in healthy individuals showed that the AHD decreased from 12.7 mm at the initial position to approximately 4.9 mm at 45° of elevation, and continued to decrease at higher angles (60°, 75°) [24]. Meanwhile, 3D–2D dynamic analyses in patients with rotator cuff tears have shown that the acromiohumeral distance is significantly smaller than in healthy individuals at various angles of arm elevation, decreasing to as little as 0.9 mm at 90° [25].

A noteworthy finding is that the static AHD (5.26 mm) in our study was already markedly lower than the normal threshold (Figure 5). Dynamic modeling further demonstrated a reduction to below 1 mm, reinforcing that these patients are at high risk for subacromial impingement and rotator cuff pathology. This has important clinical implications, as evaluating AHD in both static and dynamic conditions provides a more comprehensive understanding of the biomechanical mechanism of injury and supports decision-making for subacromial decompression in patients with rotator cuff tears.

## Conclusion

With the application of 3D technology in acromioplasty, postoperative outcomes based on objective measurements – including the Acromial Index (AI), Critical Shoulder Angle (CSA), acromiohumeral distance (AHD), and acromial morphology – were all within the normal reference ranges reported in the literature. In addition, the UCLA shoulder score demonstrated significant improvement in patients’ functional outcomes and quality of life after surgery compared to preoperative status. (Table 3).

These findings suggest that the use of 3D technology in preoperative planning for acromioplasty enables surgeons to accurately identify the optimal area for resection, estimate the appropriate amount of bone to be removed, and address the critical question of how much acromial resection is sufficient, thereby contributing to improved surgical outcomes.

This study has several limitations. First, it was a descriptive observational study combining retrospective and prospective patient enrollment, which may have introduced selection and information bias despite the use of predefined inclusion criteria and standardized data collection. Second, patients with concomitant rotator cuff tears were included in the study, and many underwent simultaneous rotator cuff repair. Therefore, postoperative improvements in pain and shoulder function may not be solely attributable to the 3D-assisted acromioplasty, as rotator cuff repair itself could have contributed to the clinical outcomes. Third, the study population was heterogeneous with respect to rotator cuff pathology, which may have influenced the postoperative results. Finally, the absence of a comparative control group prevented direct evaluation of whether preoperative 3D CT planning provides superior clinical or radiographic outcomes compared with conventional imaging-based surgical planning. Future prospective controlled studies with larger sample sizes are warranted to validate the clinical value of this technique.

## Abbreviations

AI: Acromial index
LAA: Lateral acromial angle
CSA: Critical shoulder angle
SD: Standard deviation
AHD: Acromiohumeral distance
MRI: Magnetic resonance imaging
UCLA: University of California, Los Angeles

## Data Availability

The data that support the findings of this study are not publicly available due to patient confidentiality and ethical restrictions.
